# Usability and preference of electronic vs. paper and pencil OSCE checklists by examiners and influence of checklist type on missed ratings in the Swiss Federal Licensing Exam

**DOI:** 10.3205/zma001545

**Published:** 2022-04-14

**Authors:** Felicitas L. Wagner, Sabine Feller, Felix M. Schmitz, Philippe G. Zimmermann, Rabea Krings, Sissel Guttormsen, Sören Huwendiek

**Affiliations:** 1 University of Bern, Institute for Medical Education, Department for Assessment and Evaluation, Bern, Switzerland; 2 University of Bern, Institute for Medical Education, Department for Software Development, Usability Consulting and IT Infrastructure, Bern, Switzerland; 3 University of Bern, Institute for Medical Education, Bern, Switzerland

**Keywords:** OSCE, checklists, electronic, usability, evaluation, national

## Abstract

**Background:**

Only a few studies with small sample sizes have compared electronic Objective Structured Clinical Examination (OSCE) rating checklists with traditional paper-based OSCE rating checklists. In this study, the examiner-perceived usability and preference for type of OSCE checklist (electronic vs. paper based) were compared, and the influence of OSCE checklist type on missed ratings was determined, for the Swiss Federal Licensing Examination in clinical skills for human medicine.

**Methods:**

All examiners in the Swiss Federal Licensing Examination in clinical skills for human medicine were invited over two subsequent years to evaluate the OSCE checklist type they had worked with during the examination. This was based on a questionnaire with 14 closed questions (i.e., demographic, checklist-type experience, perceived usability, checklist type preference). Furthermore, the numbers of missed ratings for the paper-based checklist were recorded.

**Results:**

The data from these examiners (*n*=377) with experience of both OSCE checklist types were analyzed. The electronic OSCE checklist was rated significantly higher on all usability aspects (i.e., ease of use, candidate rating and error correction, clarity, distraction using the checklist, overall satisfaction), except for the speed of registering comments (no significant difference). The majority of the examiners in both years (2014: 54.5%, *n*=60, 2015: 89.8%, *n*=230) reported preference for working with the electronic OSCE checklist in the future. Missed ratings were seen for 14.2% of the paper-based OSCE checklists, which were prevented with the electronic OSCE checklists.

**Conclusions:**

Electronic OSCE checklists were rated significantly more user-friendly and were preferred over paper-based OSCE checklists by a broad national sample of examiners, supporting previous results from faculty-level examinations. Furthermore, missed ratings were prevented with the electronic OSCE checklists. Overall, the use of electronic OSCE checklists is therefore advisable.

## Zusammenfassung

**Hintergrund:**

Nur wenige Studien mit kleinen Stichprobengrößen haben elektronische OSCE-Checklisten (Objective Structured Clinical Examination) mit traditionellen OSCE-Checklisten in Papierform verglichen. In dieser Studie wurden die von Prüfenden wahrgenommene Usability und Präferenz für den OSCE-Checklisten-Typ (elektronisch vs. papierbasiert) verglichen und der Einfluss des OSCE-Checklisten-Typs auf fehlende Bewertungen ermittelt für die Eidgenössische Clinical Skills-Prüfung Humanmedizin in der Schweiz.

**Methode:**

Die Prüfenden der Eidgenössischen Clinical Skills-Prüfung Humanmedizin wurden in zwei aufeinanderfolgenden Jahren gebeten, den OSCE-Checklisten-Typ zu bewerten, mit dem sie während der Prüfung gearbeitet hatten. Dies geschah anhand eines Fragebogens mit 14 geschlossenen Fragen (demographische Angaben, Erfahrung mit dem Checklisten-Typ, wahrgenommene Usability, Präferenz für den Checklisten-Typ). Außerdem wurde die Anzahl der fehlenden Bewertungen bei der papierbasierten Checkliste erfasst.

**Resultate:**

Die Daten derjenigen Prüfenden (n=377) mit Erfahrung mit beiden OSCE-Checklisten-Typen wurden ausgewertet. Die elektronische OSCE-Checkliste wurde bei allen Aspekten der Usability (einfache Benutzung, Kandidierendenbewertung und Fehlerkorrektur, Übersichtlichkeit, Ablenkung bei der Verwendung der Checkliste, Gesamtzufriedenheit) signifikant besser bewertet, mit Ausnahme der Geschwindigkeit des Erfassens von Kommentaren (kein signifikanter Unterschied). Die Mehrheit der Prüfenden in beiden Jahren (2014: 54.5%, n=60, 2015: 89.8%, n=230) gab an, in Zukunft lieber mit der elektronischen OSCE-Checkliste arbeiten zu wollen. Bei 14.2% der papierbasierten OSCE-Checklisten wurden fehlende Bewertungen festgestellt, welche mit elektronischen OSCE-Checklisten vermieden werden konnten.

**Schlussfolgerungen:**

Elektronische OSCE-Checklisten wurden von einer breiten nationalen Stichprobe von Prüfenden als deutlich benutzerfreundlicher eingestuft und gegenüber OSCE-Checklisten auf Papier bevorzugt, was frühere Ergebnisse von Prüfungen auf Fakultätsebene bestätigt. Außerdem wurden mit den elektronischen OSCE-Checklisten fehlende Bewertungen vermieden. Insgesamt ist die Verwendung elektronischer OSCE-Checklisten daher empfehlenswert.

## Background

The administration of Objective Structured Clinical Examinations (OSCEs) [1] has become the standard method to reliably assess practical skills in medical education [2], [3], [4], [5]. In an OSCE, the clinical and communication skills of the students are probed while they complete multiple stations, each of which requires them to work on a particular medical problem. During the OSCE, their performance is observed and rated by examiners through standardized, structured checklists.

Traditionally, paper-based checklists have been used to rate the candidates during an OSCE, while more recently, with the emergence of mobile electronic devices, electronic OSCE checklists have been developed and successfully implemented [6], [7], [8]. Electronic OSCE checklists are usually presented on “tablet” computers, as these are more flexible than laptops or desktop devices [7]. To date, however, few studies have compared electronic vs. paper-based OSCE checklists regarding their usability and the examiner checklist type preference. As examiners are an important stakeholder group and the users of the checklists, it is important what their usability perceptions and preferences are. According to Petersen [9], “the underlying construct of usability is to make objects compatible” with the user. Objects can be hardware, software, or every-day tools. Their design should not hinder the completion of the tasks they were designed for. Petersen further describes usability as how easy to use an object is and underlines the importance of the quality of the generated output for which an object has been created to determine its usability. OSCEs create a high cognitive load [10] for the examiners. Examiners have to simultaneously observe and rate the performances of the candidates, find the respective items in the OSCE checklist, decide on the amount of points the candidates receive for the different checklist items, and also handle the OSCE checklist. Paper-based OSCE checklists, e.g., can be several pages long [6]. Furthermore, the examination times are usually long. Considering these aspects, the OSCE checklists need to be easy to use and allow the exminers to rate candidates in an efficient and effective way to not increase the cognitive load and hinder the completion of the task they were created for, that is, rating the performances of the candidates. It is therefore important that they do not distract the examiners from their observing and rating of the performances of the candidates during examinations. Finally, also the output quality is an important aspect in the context of usability [9]. Paper-based OSCE checklists can have specific issues regarding the quality of the data. These often contain a substantial number of missed ratings, as checklist items are easily overlooked during an examination, which makes time-consuming manual data verification necessary [6]. Furthermore, paper-based checklists usually have to be scanned to transfer the OSCE results into a digital form, as a necessary step to make the results available for analysis. The use of electronic OSCE checklists on the other hand leads to better data quality because missed ratings can be prevented. Furthermore, the data can be downloaded and stored electronically directly following the examination, making the preparation of the data for analysis less error-prone and time-consuming compared to paper-based OSCE checklists [6].

A systematic review in 2019 [11] mentioned three studies that investigated examiner perceptions of electronic OSCE checklists, although only two of these [6], [12] investigated the perceived usability of paper-based versus electronic OSCE checklists. Along with studies by Hochlehnert et al. [7] and Currie et al. [8], these two studies [6], [12] suggested that the perceived usability of the respective electronic OSCE checklist tools that were used was high. However, these involved only small to medium numbers of examiners (n=10 [6], n=35 and 33 [7], n=93 [8], n=43 [12]) who were from a single faculty, thus limiting the generalizability of their results. Furthermore, as described above, one critical issue with paper-based OSCE checklists are missed ratings. However, to date little is known about the size of this problem. To our knowledge, only three studies [6], [8], [13] have addressed this problem to date.

Our research questions therefore were:

1. How does a large group of examiners from five different faculties, experienced with paper-based and electronic OSCE checklists, rate the usability of electronic versus paper-based OSCE checklists in a national licensing skills examination?
2. Which type of checklist (electronic vs. paper-based) is preferred by the examiners of a national licensing skills examination?
3. How large is the amount of missed ratings in the paper-based OSCE checklists?

## Methods

### Setting

The Swiss Federal Licensing Examination in clinical skills (which follows the principles of the OSCE examination) contained 12 different OSCE stations in both 2014 and 2015. The examination was conducted over three consecutive days at five faculties of Human Medicine in Basel, Bern, Geneva, Lausanne and Zurich. 

The paper-based OSCE checklists that were used for the 2014 examination were created using a teleform information capture system (Cardiff Software). These checklists had to be scanned after the examination, to make the results available for analysis (DR-6050 C image formula scanner; Canon). The paper-based OSCE checklists contained between 25 and 38 items, which was due to the different contents of the individual stations. These items were always sorted according to the same four dimensions, in the following order:

1. history taking,
2. physical examination,
3. management, and
4. communication.

The paper-based OSCE checklists further contained two global ratings for overall impression. At the top of the paper-based OSCE checklists, a reminder sentence was included stating that all of the items in the checklist had to be completed.

**Figure 1:**
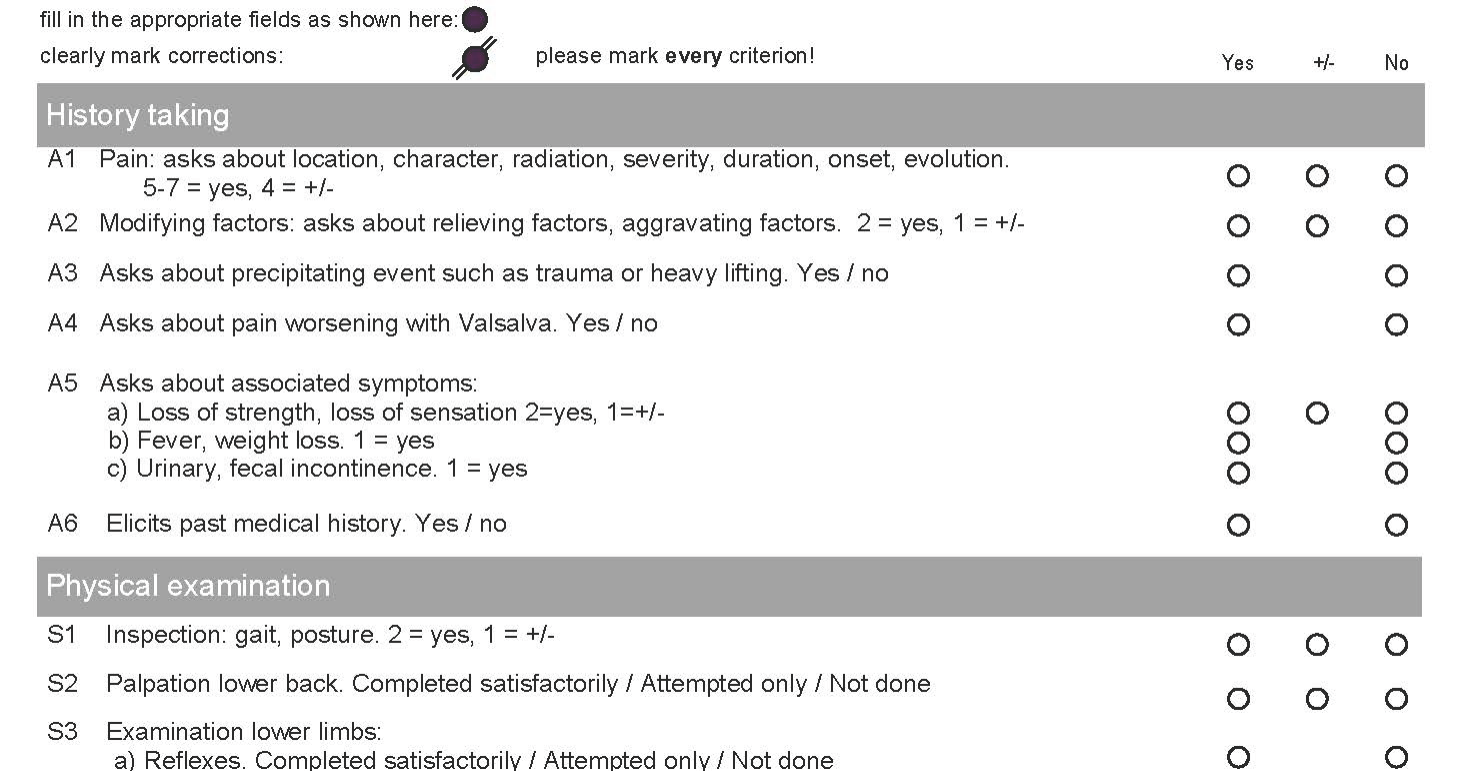
Example image of part of the paper-based OSCE checklist (fictitious candidate)

**Figure 2:**
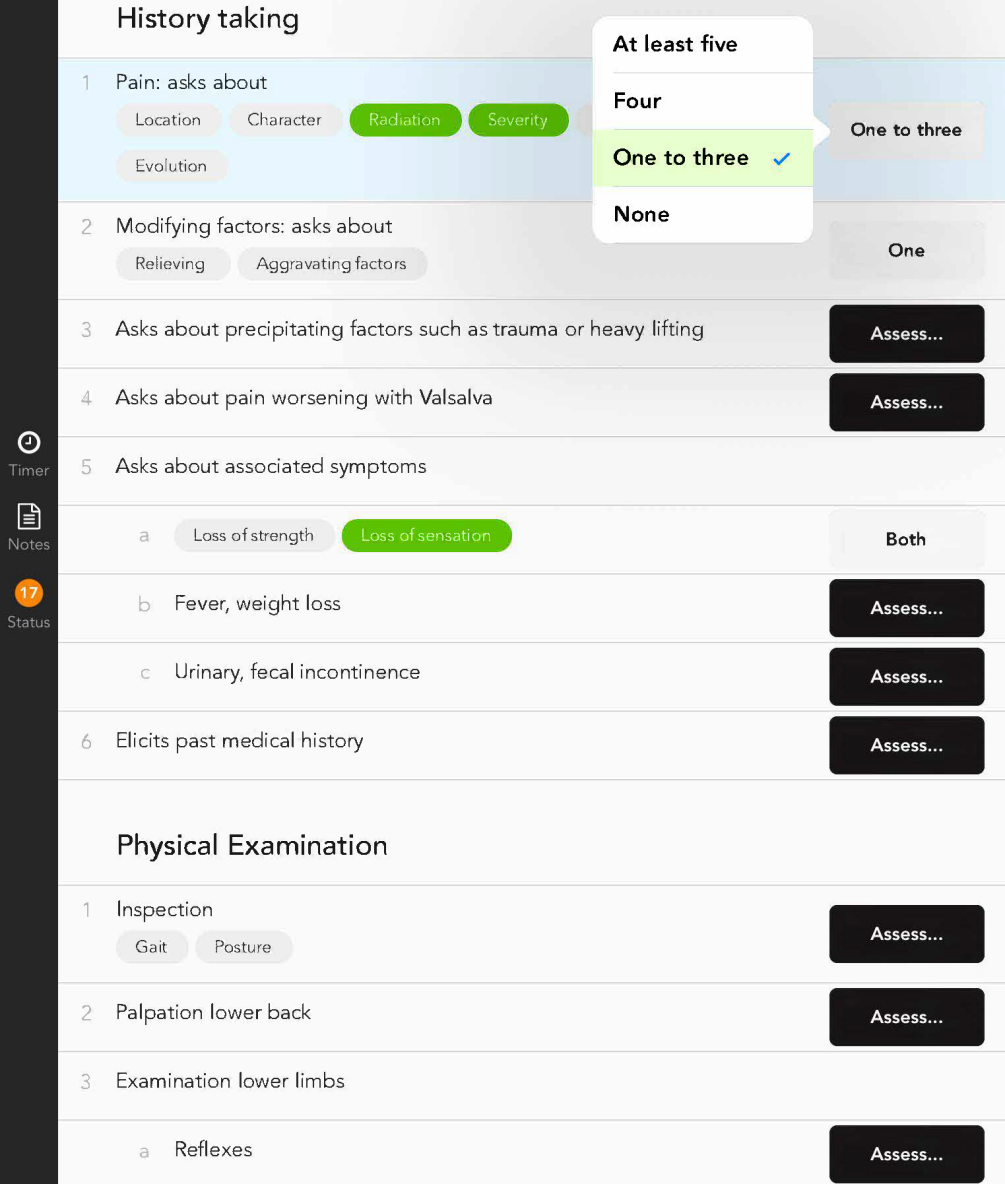
Example image of part of the electronic OSCE checklist (fictitious candidate)

The electronic OSCE checklists in the 2015 examination were completed using identical fourth generation iPad devices (2014, Apple). The iPad app “OSCE-Eval” [https://eosce.ch/], version 2.1, was used to rate the candidates. This app includes different features to reduce potential errors and to ensure that the collected data are readily available for subsequent analyses. The users receive visual feedback to make sure that every item is completed. Every item had to be evaluated to finally submit the checklist. After the final assessment, no more changes were possible. Encrypted data were synchronized with a secure server, and the data were automatically exported to an electronic spreadsheet. Depending on the specific content of the stations, the electronic OSCE checklists contained between 21 and 42 items. The items were sorted according to the same four dimensions as in the paper-based OSCE checklist. In addition, there were two global ratings in each checklist for overall impressions (see figure 1 (Fig. 1) and figure 2 (Fig. 2) for examples of both checklist types). The electronic checklists were developed according to the usability standards at the time, and considerable effort was invested to iteratively check their acceptance during their development.

In the introduction to the OSCE exam in both 2014 and 2015, the examiners were briefly instructed on how to use the checklists and how to rate the candidates. Additionally, the examiners working with the electronic OSCE checklist (in 2015) had the possibility to watch a specific, 6-min training video before the examination, to familiarize themselves with the checklist and the software. The training video was the same that was used by Schmitz et al. [6], who reported that the video was comprehensible and effective. 

#### Sample

In 2014, 696 examiners rated the performances of the candidates at the different stations using the paper-based OSCE checklist. In 2015, 696 examiners rated the performances of the candidates at the different stations using the electronic OSCE checklist. All examiners were invited to take part in the survey. Only data from examiners who had worked with both checklist types (paper-based and electronic) at least one time have been included to allow for comparisons between the electronic and the paper-based OSCE checklist. 

#### Material

The examiners evaluated the OSCE checklist type (i.e., paper-based in 2014, electronic in 2015) that they had used to rate the candidates through completion of a questionnaire (see table 1 (Tab. 1)). The questionnaire contained a total of 14 closed questions: demographic information (three questions); experience with touch-screen devices and the two different OSCE checklist types (electronic, paper-based; three questions); subjective usability (seven questions); and future checklist type preference (one question). The replies were based on a 7-point Likert scale, from 1, as “do not agree at all”, to 7, as “fully agree”. 

The usability questions were informed by System Usability Scale [14]. However, the questionnaire had only seven questions regarding usability, to reduce the efforts of the examiners, and six further questions concerning the specific requirements in an OSCE examination setting (e.g., questions regarding speed of rating the candidates, distraction from observing the candidates, speed of correcting input errors). These questions further reflect usability aspects as indicated by Petersen [9] (ease of use, not hindring the completion of the task).

The questionnaire was available in German and French. To ensure that the questions were clear, think-aloud trials [15] with three examiners were conducted in both languages. During each think-aloud, the participants were asked to verbalize their thoughts while they were working on the questionnaire. This method thus allowed the identification of ambiguities and misconceptions, and it was used to make sure that the questions were correctly understood by the participants. The same questionnaire was used in 2014 and 2015. 

Finally, to determine the completeness of the data, the number of missed ratings for the paper-based checklists was recorded. 

#### Procedure

For both years, within the first week following the examination, all of the examiners were invited to evaluate the OSCE checklist type they had worked with during the examination (i.e., paper-based in 2014, electronic in 2015). Hence, a comparison across the two OSCE checklist types was possible. In 2014, the examiners received the questionnaire in paper-based form directly after the exam, in 2015 the questionnaire was implemented online using the online survey tool “Unipark” [https://www.unipark.com/] and the examiners were invited via e-mail. This type of study was regarded as exempt from formal ethical approval according to the regulations of the Ethics Committee (“Kantonale Ethikkommission” Canton of Bern) associated with the Medical Faculty of the University of Bern. Participation in the survey was voluntary and anonymous and participants have not been exposed to any conceivable risks by participating in this study. 

#### Statistical analysis

Frequencies were analysed to report demographics of the sample. For comparisons between the paper-based and the electronic OSCE checklist, Mann-Whitney-Tests were used as the data are ordinally scaled. The alpha level was adjusted for multiple testing according to Bonferroni. The effect size is given by Pearson’s r, the magnitude of the effects was judged according to Cohen [16]. 

## Results

### Participants

In 2014, 696 examiners rated the performances of the candidates at the different stations using the paper-based OSCE checklist. Of these, 540 (78%) completed the survey questionnaire. In 2015, 696 examiners rated the performances of the candidates at the different stations using the electronic OSCE checklist. Here, the response rate was 58%, where 406 of the 696 examiners completed the survey questionnaire. 

The questionnaire datasets from 35 (4%) examiners were removed from the database due to missing data (2014: 19 datasets; 2015: 16 datasets). All datasets that were removed contained no useful information as these persons stopped filling out the questionnaire after the language selection right in the beginning of the questionnaire. Datasets from 911 (96%) examiners were taken forward for the analysis. Of these 911 examiners, 377 (41%) reported that they had worked with both checklist types (paper-based and electronic) at least once. This included examiners from both years, as examiners who worked with the paper-based OSCE checklist in 2014 might have had previous experience with electronic OSCE checklists for other examinations. Only the responses from these examiners who had experience with both checklist types were included in the quantitative analysis. Table 2 (Tab. 2) details the experiences of the examiners for both of the checklist types. 

#### Demographic questions

The demographics of the examiners in the final sample are presented in table 2 (Tab. 2). 

#### Touch-screen experience

Most examiners from both years had vast (M_2014_=6.21, M_2015_=6.08; scale: 1, “not experienced at all”, to 7, “very experienced”) and comparable experience with the use of touch-screens (*U*=13933.5, *n.s.*). Thus, the examiner ratings and checklist type preferences (i.e., paper-based vs. electronic) are unlikely to be influenced by their prior experience with touch-screen devices. 

#### Usability 

Mann-Whitney-Tests indicated that the majority of the examiner’s usability ratings significantly differed between the electronic and the paper-based OSCE checklists (see table 3 (Tab. 3)). The Bonferroni adjusted alpha level for the seven comparisons described in the following was .007. The electronic OSCE checklist was rated as significantly easier to use (*U*=9799.5, *p*<0.007, *r*=.33) and clearer (*U*=11441.5, *p*<0.007, *r*=.21) than the paper-based OSCE checklist. The electronic OSCE checklist also scored significantly higher for speed of rating of candidates (*U*=11408, *p*<0.007, *r*=.20) and correcting input errors (*U*=7766.5, *p*<0.007, *r*=.41). Furthermore, filling in the electronic OSCE checklist was rated as significantly less distracting than filling in the paper-based OSCE checklist (*U*=11673, *p*<0.007, *r*=.18). The speed of writing comments was not rated differently between the two checklist types (*U*=12554, *p*=0.035, n.s., *r*=.11). The overall satisfaction of examiners was also significantly higher with the electronic than the paper-based OSCE checklist (*U*=7499, *p*<0.007, *r*=.42) (see table 4 (Tab. 4)). The effects sizes according to Cohen [16] were either medium (between 0.3 and 0.5), or low (between 0.1 and 0.3). Taken together, the electronic OSCE checklist received higher usability ratings than the paper-based OSCE checklist.

#### Checklist type preference

At the end of the questionnaire, the examiners from both 2014 and 2015 who had indicated experience with both electronic and paper-based OSCE checklists were asked whether they would rather work with electronic or paper-based OSCE checklists in the future (see table 4 (Tab. 4)). Across both years, the majority of these examiners who had had experience with both electronic and paper-based OSCE checklists preferred the electronic format. 

#### Missed ratings for the paper-based OSCE checklist

The number of missed ratings in the paper-based checklist was recorded (see table 5 (Tab. 5)). In 1428 (14.2%) of all of the paper-based checklists, there were missed ratings. These checklists contained between 1 and 12 missed ratings (*M*=1.45, *SD*=1.03). In the majority of these checklists, one rating was missed. 

## Discussion

In this study that included a large sample of examiners experienced with electronic and paper-based OSCE checklists from five different faculties in the Swiss Federal licensing examination over two subsequent years, the perceived usability was significantly higher for the electronic than the paper-based OSCE checklist in all aspects except for the speed of writing comments, where no significant difference was observed. Furthermore, the vast majority of the examiners preferred to work with the electronic OSCE checklist in the future. Finally, there were missed ratings in 14.2% of all of the paper-based checklists versus none in the electronic checklist (as the electronic system required all checklist items to be completed). 

We were able to show here that electronic OSCE checklists have higher perceived usability than paper-based checkists across a large dataset of examiners from five different faculties in the Swiss Federal Licensing examination. To date, the usability of electronic OSCE checklists has only been investigated in studies that have included mostly small to medium numbers of examiners [6], [7], [8], [12]. Given the high cognitive load [10] that these examiners experience during an OSCE, the usability of the OSCE checklist type to rate the candidates is essential. The examiners in this study overall were more satisfied with the electronic than with the paper-based OSCE checklist. They perceived the electronic OSCE checklist as clearer and easier to use compared to the paper-based OSCE checklist, while they also felt less distracted during the examinations. Furthermore, rating the candidates as well as correcting input errors was reported to be easier for the electronic compared to the paper-based OSCE checklist. However, there was no significant difference regarding the perceived speed of writing comments on a touch-pad (i.e., the electronic OSCE checklist) and writing comments on paper. It can be assumed that all of these factors contribute to allow the examiners to better focus their attention on the candidates and their performances during the examinations, thus keeping the cognitive load of the examiners lower in the electronic OSCE checklist.

Our analysis of missed ratings in the paper-based OSCE checklists showed that in some of the individual checklists, there were indeed a large number of missed items. However, overall, the amount of missed items was moderate, as this was seen for 14.2% of all of the paper-based OSCE checklists. In the majority of these (10.6% of all checklists containing missed items), only one missed item was found. The mean number of missed ratings in these checklists was 1.45 overall, which is a good deal lower compared to the missing ratings reported by Schmitz et al. [6]. The number of missed ratings for such paper-based checklists therefore appears to vary greatly between different examinations and examiners. With the use of electronic OSCE checklists on the other hand, missed ratings can be prevented altogether, which represents an important advantage of the electronic OSCE checklist, as it ensures higher data quality [6], [8], [12]. The output quality as an important aspect of usability [9] thus is clearly higher in electronic OSCE checklists. Despite the introduction to the paper-based OSCE checklist at the beginning of the exam and a reminder sentence on top of the checklist, there were still cases where examiners missed to rate items. One possible explanation could be that because of the lower usability of the paper-based checklist, the coginitve load became too high for the examiners in some situations which may have lead to the missed ratings. 

The analysis of the checklist type preferences showed a clear result. Both in 2014 (54.5%) and in 2015 (89.9%) the majority of the examiners who were experienced with both OSCE checklist types specified that they would prefer to work with electronic OSCE checklists in the future, thus confirming the results of Schmitz et al. [6] and Currie et al. [8]. We assume that this finding is related to the higher perceived usability of the electronic OSCE checklist. 

The strengths of the present study are the large numbers of examiners involved, the high proportion of these examiners who had experience with both types of OSCE checklists (i.e., paper-based and electronic), and the multi-institutional (national) setting that included examiners from five different faculties from two language regions. 

A limitation of this study is that except for the missed ratings, these data are based on the subjective impressions of the examiners with regard to the use of these checklists, rather than providing an objective measure of usability.

## Conclusions

The results of the present study show that the perceived usability of electronic OSCE checklists compared to traditional paper-based checklists was significantly higher, except for the speed of writing comments, where there was no statistically significant difference. The majority of the examiners experienced with both electronic and paper-based OSCE checklists preferred to work with the electronic OSCE checklists in the future. Importantly, while missed ratings are a common issue in paper-based checklists, they can be completely prevented when electronic OSCE checklists are used, which ensures high data quality. Overall, the results of the present study with a large number of examiners across different faculties show that if developed with a focus on usability, electronic OSCE checklists have advantages that go beyond technical factors, and so they can be considered as an advisable alternative to paper-based OSCE checklists. 

## Declarations

### Ethics approval and consent to participate

This type of study was regarded as exempt from formal ethical approval according to the regulations of the Ethics Committee (‘Kantonale Ethikkommission’ Canton of Bern) associated with the Medical Faculty of the University of Bern. Nevertheless, we confirm that this study was conducted according to the Declaration of Helsinki where applicable for this type of study, and that participants took part on a voluntary basis, they cannot be identified by the material presented, and they have not been exposed to any conceivable risks by participating in this study.

#### Consent for publication

Not applicable. The images provided in this manuscript show a fictitious example created specifically for this publication to illustrate the electronic and paper-based OSCE checklists.

#### Availability of data and materials

The datasets analysed during the current study are available from the corresponding author on reasonable request. More information about the OSCE-Eval application can be found at the website [https://eosce.ch/].

## Funding

This study was funded by the Institute for Medical Education, University of Bern, Switzerland.

## Authors’ contributions

F.W. analyzed and interpreted the data and wrote the manuscript with support from S.H. R.K. helped in the analysis of the data. S.H., S.F., F.S. and P.Z. constructed the questionnaire for the survey. S.G., S.F., R.K., F.S., P.Z. and S.H. critically revised the manuscript. All authors have read and approved the final manuscript.

## Acknowledgements

We would like to thank: the examiners for participating in the survey; the site responsibles, Monica Escher, Felix Eymann, Claudia Glauser, David Gachoud and Roger Kropf; the members of the clinical skills working group, Gianmarco Balestra, Christoph Berendonk, Sylvie Félix, Sabine Feller, Philippe Huber, Matteo Monti, Kai Schnabel, and Ernst Jünger; and the IT support team of the Swiss Federal Licensing Examination at the faculties of Basel, Bern, Geneva, Lausanne and Zurich. Furthermore, we thank Daniel Stricker for his advice on the analysis of the missed items. Finally, we would like to thank the Federal Office of Public Health and the Examinations Commission for supporting this examination. 

## Competing interests

The authors declare that they have no competing interests. 

The OSCE-Eval application that was used to implement the electronic OSCE checklist was designed and developed by the Department for Software Development, Usability Consulting and IT Infrastructure (ASCII), at the Institute for Medical Education. More information about the application can be accessed through the website [https://eosce.ch/].

## Figures and Tables

**Table 1:**
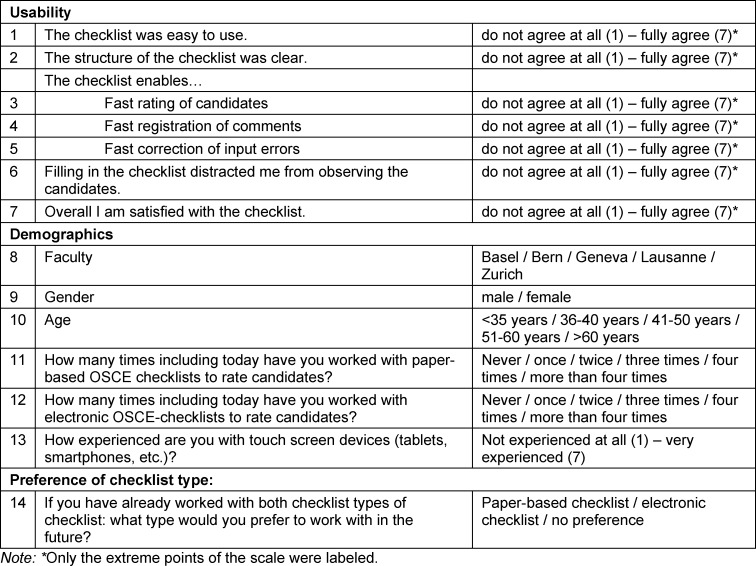
Evaluation questionnaire. The questions have been translated from German and French to English for clarity here.

**Table 2:**
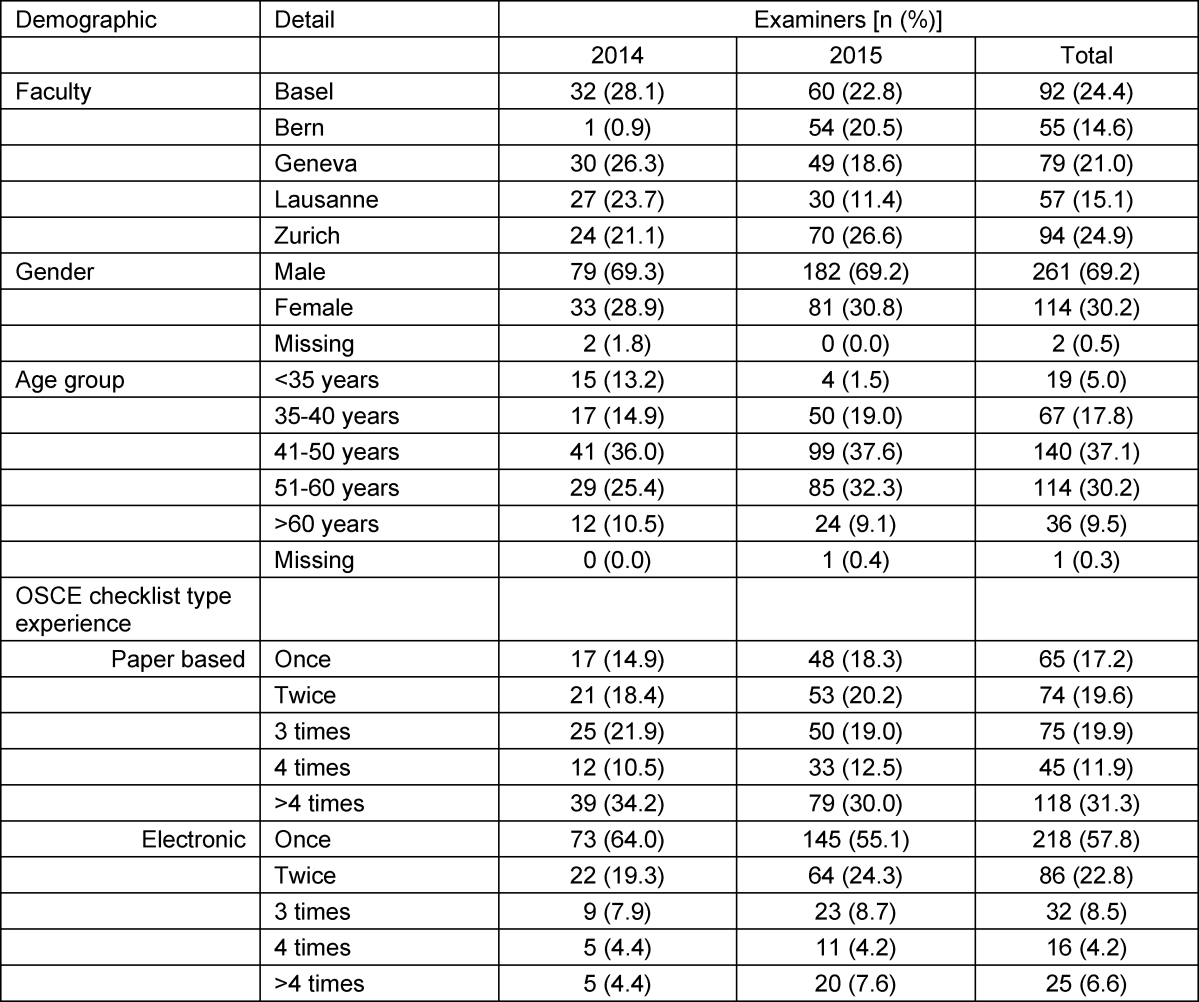
Demographics of the final study sample (examiners experienced with both the electronic and the paper-based OSCE checklist types)

**Table 3:**
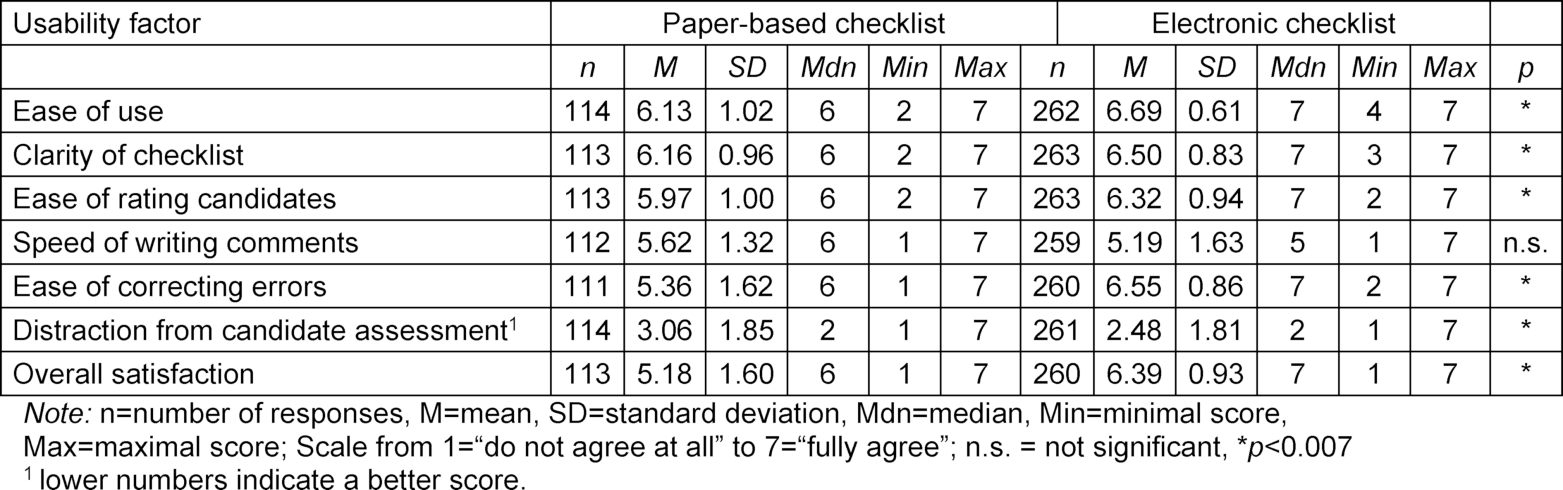
Usability ratings of the electronic and paper-based OSCE checklists

**Table 4:**
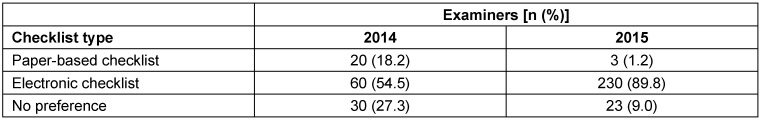
Future preference for use of the OSCE checklist type

**Table 5:**
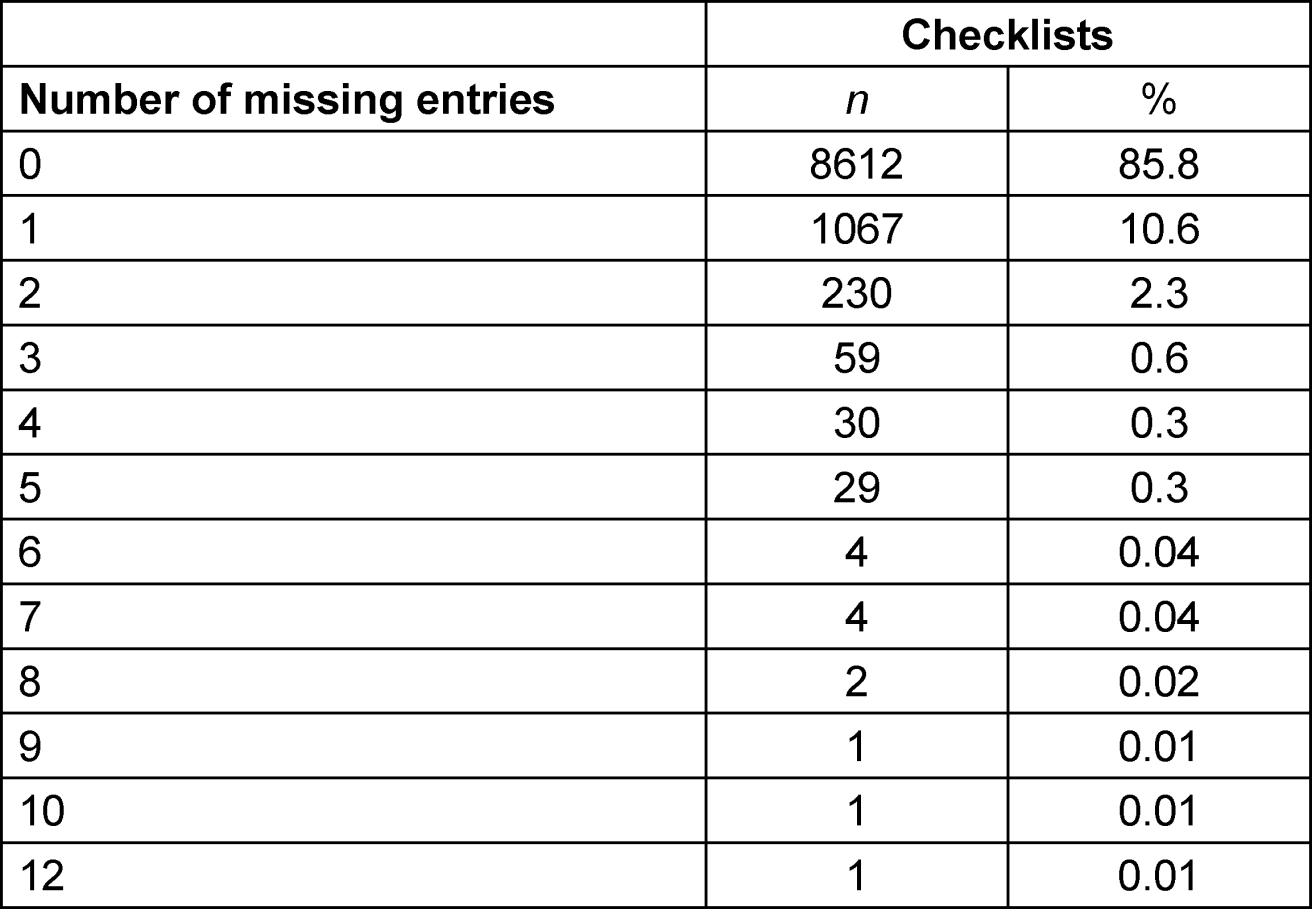
Number of missings entries for the paper-based OSCE checklists
